# Induction of p53-Independent Apoptosis and G1 Cell Cycle Arrest by Fucoidan in HCT116 Human Colorectal Carcinoma Cells

**DOI:** 10.3390/md15060154

**Published:** 2017-05-30

**Authors:** Hye Young Park, Shin-Hyung Park, Jin-Woo Jeong, Dahye Yoon, Min Ho Han, Dae-Sung Lee, Grace Choi, Mi-Jin Yim, Jeong Min Lee, Do-Hyung Kim, Gi-Young Kim, Il-Whan Choi, Suhkmann Kim, Heui-Soo Kim, Hee-Jae Cha, Yung Hyun Choi

**Affiliations:** 1 Department of Biochemistry, Dong-Eui University College of Korean Medicine, Busan 47227, Korea; nattier2895@naver.com; 2 Department of Pathology, Dong-Eui University College of Korean Medicine, Busan 47227, Korea; omdpark@deu.ac.kr; 3 Anti-Aging Research Center and Blue-Bio Industry RIC, Dongeui University, Busan 47227, Korea; jinwooyo@nate.com; 4 Department of Chemistry, College of Natural Sciences, Pusan National University, Busan 46241, Korea; dahyenim89@gmail.com (D.Y.); suhkmann@pusan.ac.kr (S.K.); 5 Department of Applied Research, National Marine Biodiversity Institute of Korea, Seocheon 33662, Korea; mhhan@mabik.re.kr (M.H.H.); daesung@mabik.re.kr (D.-S.L.); gchoi@mabik.re.kr (G.C.); mjyim@mabik.re.kr (M.-J.Y.); lshjm@mabik.re.kr (J.M.L.); 6 Department of Aquatic Life Medicine, Pukyong National University, Busan, 48513, Korea; dhkim@pknu.ac.kr; 7 Laboratory of Immunobiology, Department of Marine Life Sciences, Jeju National University, Jeju 63243, Korea; immunkim@jejunu.ac.kr; 8 Department of Microbiology, College of Medicine, Inje University, Busan 47392, Korea; cihima@inje.ac.kr; 9 Department of Biological Sciences, College of Natural Sciences, Pusan National University, Busan 46241, Korea; khs307@pusan.ac.kr; 10 Departments of Parasitology and Genetics, Kosin University College of Medicine, Busan 49267, Korea

**Keywords:** fucoidan, colorectal carcinoma, p53, G1 arrest, apoptosis

## Abstract

It is well known that fucoidan, a natural sulfated polysaccharide present in various brown algae, mediates anticancer effects through the induction of cell cycle arrest and apoptosis. Nevertheless, the role of tumor suppressor p53 in the mechanism action of fucoidan remains unclear. Here, we investigated the anticancer effect of fucoidan on two p53 isogenic HCT116 (p53+/+ and p53−/−) cell lines. Our results showed that inhibition of cell viability, induction of apoptosis and DNA damage by treatment with fucoidan were similar in two cell lines. Flow cytometric analysis revealed that fucoidan resulted in G1 arrest in the cell cycle progression, which correlated with the inhibition of phosphorylation of retinoblastoma protein (pRB) and concomitant association of pRB with the transcription factor E2Fs. Furthermore, treatment with fucoidan obviously upregulated the expression of cyclin-dependent kinase (CDK) inhibitors, such as p21WAF1/CIP1 and p27KIP1, which was paralleled by an enhanced binding with CDK2 and CDK4. These events also commonly occurred in both cell lines, suggesting that fucoidan triggered G1 arrest and apoptosis in HCT116 cells by a p53-independent mechanism. Thus, given that most tumors exhibit functional p53 inactivation, fucoidan could be a possible therapeutic option for cancer treatment regardless of the p53 status.

## 1. Introduction

Colorectal cancer (CRC) is one of the most common cancers worldwide. It accounts for about 10% of all cancer cases, making it the third most common cancer. In addition, CRC is the fourth leading cause of cancer-related death, with more than 700,000 people dying from CRC every year [1,2,3]. Although most cases of CRC are detected in Western countries, its incidence has rapidly increased in East Asia and Eastern Europe over the past few years due to the dietary shift toward a more “Western diet” characterized by a large amount of red meat and fat [4,5]. While surgery can be curative for CRC in the early stage, treatments for later-stage patients with multiple metastases are less effective; such treatments are often directed at palliation to relieve symptoms. Thus, there is a need to develop novel therapeutic strategies for CRC [6].

Induction of apoptotic cell death or cell cycle arrest in cancer cells is thought to be the principal strategy for eliminating tumors. The tumor suppressor p53, which is considered the “master regulator” of cell fate, plays a pivotal role in the regulation of both apoptosis and the cell cycle [7,8]. p53 engages its anticancer potential through modulation of the target genes involved in DNA repair, apoptosis cascade, and cell cycle regulation, which finally contributes to the suppression of cancer progression [9,10]. However, p53 has been observed to be mutated or deleted in over 50% of human cancers, including CRC, thereby making the p53 gene a major obstacle for cancer therapy [11,12,13]. Consequently, tumor cells lacking the p53 gene generally display greater resistance to anticancer agents [8,14]. Thus, the development of ideal chemotherapeutic candidates that express their anticancer properties regardless of the p53 status in tumors could be a desirable strategy to treat cancer. 

Fucoidan is a natural fucose-containing sulfated polysaccharide that exists in various species of brown seaweed [15,16]. Numerous studies have demonstrated that fucoidan has anticancer effects, which are mainly caused by an inhibition of cancer cell growth and promotion of antitumor cytokine secretion, as well as lymphocyte proliferation [17,18]. It has also been reported that fucoidan induces apoptosis and cell cycle arrest in a variety of cancer cell types, including leukemia, hepatocellular carcinoma, prostate cancer, breast cancer and bladder cancer [19,20,21,22,23,24]. Although its cytotoxic effect in colon cancer cells has already been reported [21], it is still unclear whether apoptosis induction by fucoidan requires p53 function. Furthermore, the role of p53 in fucoidan-induced cell cycle arrest has yet to be adequately explored in colon cancer. Therefore, in the current study, we investigated the anticancer properties of fucoidan in HCT116 human colorectal carcinoma lines with different p53 status. We found that fucoidan triggered apoptosis and G1 cell cycle arrest not only in wild-type p53 (p53+/+) HCT116 cells but also in p53 allele-null (p53−/−) HCT116 cells, suggesting that fucoidan could be a possible therapeutic option for CRC regardless of its p53 status.

## 2. Results

### 2.1. Fucoidan Suppresses Cell Survival in HCT116 Cells

**Figure 1:**
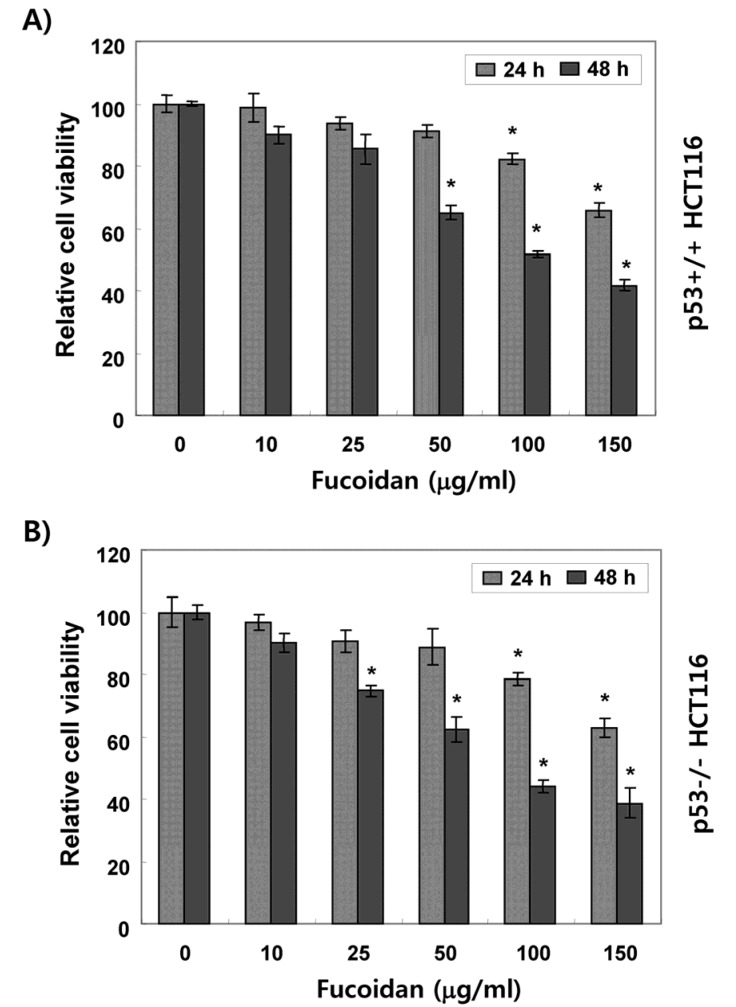
Effects of fucoidan on cell viability of HCT116 cells. p53+/+ (**A**) and p53−/− (**B**) HCT116 cells were treated with the indicated concentrations of fucoidan for 24 h or 48 h. The cell viability was evaluated by a 3-(4,5-dimethyl-2-thiazolyl)-2,5-diphenyl-2H-tetrazolium bromide (MTT) assay. Data are expressed as the mean standard deviation (±SD) of three independent experiments. Significance was determined by the Student *t*-test (* *p* < 0.05 vs. untreated control).

**Figure 2:**
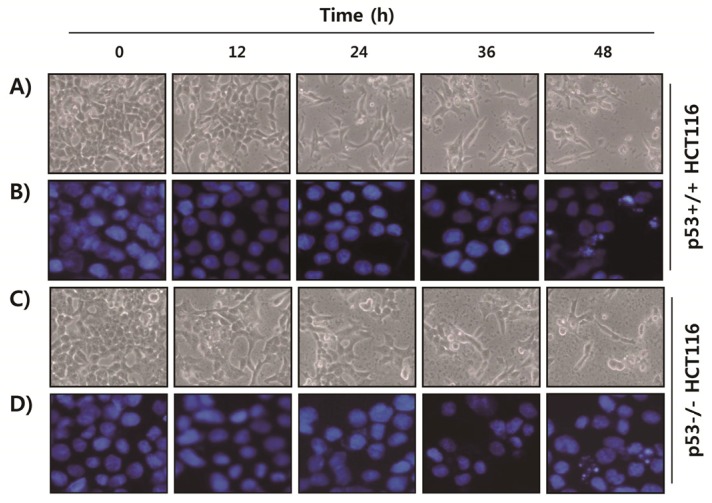
Effects of fucoidan on cell morphological changes in HCT116 cells. p53+/+ (**A**,**B**) and p53−/− (**C**,**D**) HCT116 cells were treated with 150 μg/mL of fucoidan for the indicated times. (**A**,**C**) Cell density and morphological changes were visualized using an inverted microscope (Magnification, ×200). (**B**,**D**) The cells were fixed and stained with 4′,6-diamidino-2-phenylindole (DAPI) to visualize DNA. The stained nuclei were then photographed with a fluorescence microscope (Magnification, ×400).

We first evaluated the influence of fucoidan on HCT116 cell viability by a 3-(4,5-dimethyl-2-thiazolyl)-2,5-diphenyl-2H-tetrazolium bromide (MTT) assay. As shown in Figure 1, the cell viability was gradually reduced by fucoidan treatment in a concentration- and time-dependent manner in both p53+/+ and p53−/− HCT116 cell lines. Morphological observances of p53+/+ HCT116 cells showed a decreased cell density, as well as an increased number of floating dead cells following fucoidan treatment in a time-dependent manner (Figure 2A). The results of 4′,6-diamidino-2-phenylindole (DAPI) staining showed that the nuclei of fucoidan-treated p53+/+ HCT116 cells were more condensed and fragmented than those of control cells, which usually occurs in apoptotic cell death (Figure 2B). These morphological changes by fucoidan were observed to be approximately the same in p53−/− HCT116 cells as well (Figure 2C,D).

### 2.2. Fucoidan Induces Apoptotic Cell Death in HCT116 Cells

**Figure 3:**
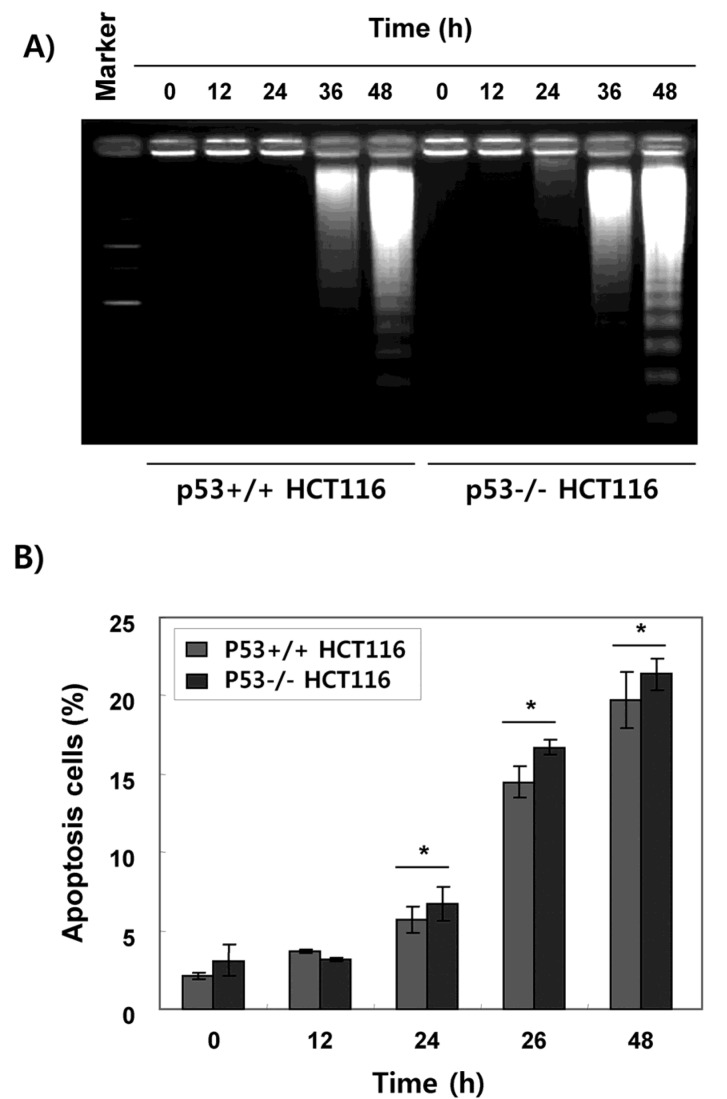
Induction of apoptosis by fucoidan in HCT116 cells. p53+/+ and p53−/− HCT116 cells were treated with 150 μg/mL fucoidan for various times. (**A**) The cells were collected, and the extracted fragmented DNA was separated on 1.5% agarose gel and visualized under ultraviolet (UV) light by ethidium bromide (EtBr) staining. (**B**) The cells were stained with propidium iodide (PI) for DNA flow cytometry analysis. Percentages of apoptotic cells were determined by counting the sub-G1 phase cell population. Data are expressed as the mean ± SD of three independent experiments. Significance was determined by the Student *t*-test 0.0 (* *p* < 5 vs. untreated control).

Since DAPI staining is an indirect method of evaluating apoptosis, we further conducted DNA fragmentation assay. As shown in Figure 3A, the fragmented DNA was markedly increased after fucoidan treatment in both p53+/+ and p53−/− HCT116 cells (Figure 3A). Flow cytometry analysis also showed a time-dependent increase of apoptotic cells, represented by the sub-G1 phase cell accumulation, regardless of the p53 status in HCT116 cells (Figure 3B).

### 2.3. Fucoidan Enhances Degradation of Poly(ADP-ribose) Polymerase (PARP) and Phosphorylation of γH2AX in HCT116 Cells

**Figure 4:**
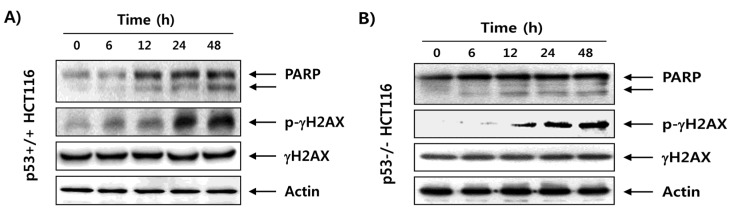
Degradation of PARP and phosphorylation of γH2AX by fucoidan in HCT116 cells. After treatment with 150 μg/mL of fucoidan for the indicated times, the cells (**A**, p53+/+ HCT116; **B**, p53−/− HCT116) were lysed; then, equal amounts of proteins were separated on sodium dodecyl sulfate (SDS)–polyacrylamide gels and transferred onto membranes. Membranes were probed with the indicated antibodies, and the proteins were visualized by an enhanced chemiluminescence (ECL) detection system. Actin was used as an internal control.

Since the degradation of PARP protein is used as representative evidence that one of the proteins is fragmented by the activated caspase cascade [25], we next examined the effect of fucoidan on the expression of PARP to provide further support for the hypothesis that fucoidan-induced apoptosis in HCT116 cells. Our immunoblotting results indicated that there was a marked increase in the levels of cleaved PARP (85 kDa) expression in fucoidan-treated p53+/+ and p53−/− HCT116 cells compared with the control (Figure 4). We also investigated the effects of fucoidan on the expression of γH2AX to address whether the inhibition of proliferation of HCT116 cells by fucoidan is associated with DNA damage induction. As shown in Figure 4, our data revealed that exposure of HCT116 cells to fucoidan increased the phosphorylation of histone γH2AX on serine 139, a marker of DNA double strand breaks [26]. However, total levels of γH2AX protein were relatively unaffected by treatment with fucoidan, and these effects were similar in p53+/+ and p53−/− HCT116 cells.

### 2.4. Fucoidan Induces G1 Cell Cycle Arrest in HCT116 Cells

**Figure 5:**
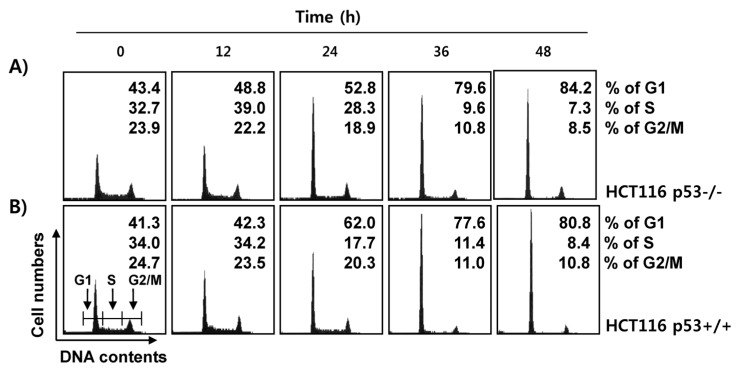
Effects of fucoidan on cell cycle distribution in HCT116 cells. p53+/+ and p53−/− HCT116 cells were treated with fucoidan (150 μg/mL) for the indicated time periods. The cells were collected and stained with PI for cell cycle analysis using a flow cytometer. Data are expressed as the means of three independent experiments.

Next, we investigated whether induction of apoptosis by fucoidan was associated with cell cycle arrest. As demonstrated in Figure 5, each phase of the cell cycle showed a normal distribution in the control p53+/+ and p53−/− HCT116 cells. However, following exposure to fucoidan for 48 h, the number of cells corresponding to the G1 phase in p53+/+ HCT116 cells was doubled, while the numbers of cells in the S and G2/M phases were decreased in a time-dependent manner. The fucoidan-treated p53−/− cells showed a similar pattern to the p53+/+ cells, suggesting that fucoidan-induced cell cycle arrest in the G1 phase was independent of p53 expression.

### 2.5. Fucoidan Inhibits pRB Phosphorylation and Enhances Binding pRB with E2Fs in HCT116 Cells

**Figure 6:**
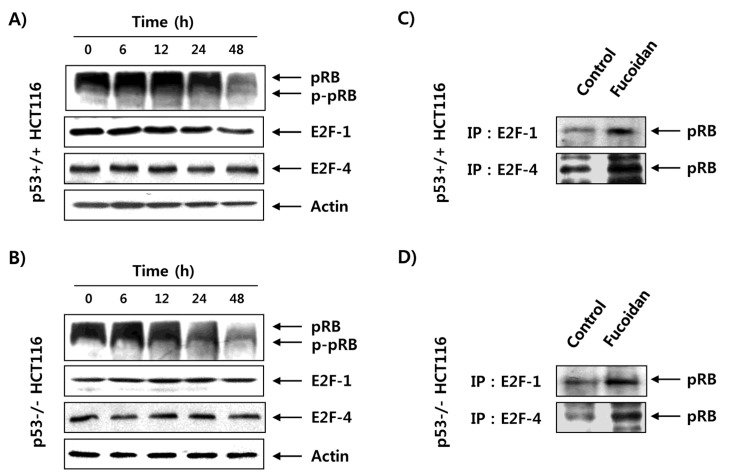
Inhibition of pRB phosphorylation and increase of pRB/E2F complexes by fucoidan in HCT116 cells. p53+/+ (**A**,**C**) and p53−/− (**B**,**D**) HCT116 cells were treated with 150 μg/mL of fucoidan for the indicated times. (**A**,**B**) The cells were lysed, and then equal amounts of proteins were separated on SDS–polyacrylamide gels and transferred onto membranes. Membranes were probed with the indicated antibodies, and the proteins were visualized by an enhanced chemiluminescence (ECL) detection system. Actin was used as an internal control. (**C**,**D**) The cells were incubated with or without 150 μg/mL of fucoidan for 24 h, and then equal amounts of proteins (500 μg) were immunoprecipitated with the anti-E2F-1 or anti-E2F-4 antibodies. Immunocomplexes were separated by 8% SDS–polyacrylamide gel electrophoresis (PAGE), transferred to membranes, and probed with the anti-pRB antibodies.

To uncover the mechanism of fucoidan-triggered G1 cell cycle arrest, we investigated fucoidan’s influence on the expression of retinoblastoma protein (pRB) and transcription factor E2F family proteins, which function as opposing molecules that control the G1/S transition [27,28]. The immunoblotting results indicated that the expression of phosphorylated pRB in p53+/+ HCT116 cells was decreased by fucoidan treatment in a time-dependent manner without affecting expression of E2Fs, such as E2F-1 and E2F-4 (Figure 6A). To investigate the influence of fucoidan on the interaction between pRB and E2Fs, we conducted coimmunoprecipitation. The results showed that fucoidan treatment markedly strengthened the association between pRB and E2Fs in p53+/+ cells, suggesting that the dephosphorylated pRB strongly bound to E2Fs (Figure 6C). This phenomenon was consistently observed in p53−/− HCT116 cells (Figure 6B,D).

### 2.6. Fucoidan Regulates the Expression of G1 Phase-Associated Cyclins and CDKs in HCT116 Cells

**Figure 7:**
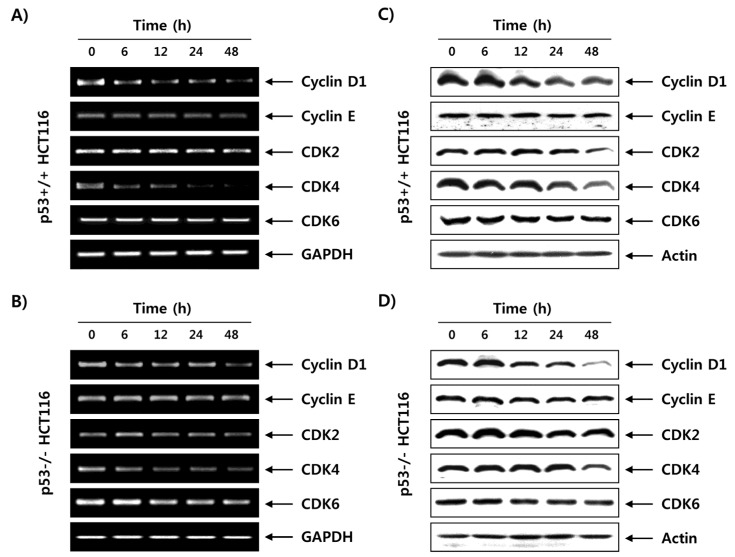
Effects of fucoidan on the expression of cyclins and cyclin-dependent kinases (CDKs) in HCT116 cells. p53+/+ (**A**,**C**) and p53−/− (**C**,**D**) HCT116 cells were treated with 150 μg/mL of fucoidan for various times. (**A**,**B**) The total RNA was extracted from the cells, and the mRNA levels of cyclins and CDKs were examined by reverse transcription–polymerase chain reaction (RT–PCR). Glyceraldehyde 3-phosphate dehydrogenase (GAPDH) was used as an internal control. (**C**,**D**) The cells were lysed, and then equal amounts of proteins were separated on SDS–polyacrylamide gels and transferred onto membranes. Membranes were probed with the indicated antibodies, and the proteins were visualized using an ECL detection system. Actin was used as an internal control.

Because the activities of G1-phase cyclin-dependent kinases (CDKs), such as CDK2, CDK4 and CDK6, are positively regulated by bound cyclins, such as D-type cyclins (cyclins D1, D2 and D3) and cyclin E [27,28], the expression of G1/S cell cycle progression-related genes in response to fucoidan treatment was next determined. As shown in Figure 7A,B, the mRNA expressions of cyclin D1, CDK2 and CDK4 were partially decreased by fucoidan treatment in a time-dependent manner in both the p53+/+ and p53−/− HCT116 cell lines, whereas the expression levels of cyclin E and CDK6 remained unchanged. In accordance with the transcriptional regulation, fucoidan downregulated the levels of cyclin D1, CDK2 and CDK4 proteins, but did not affect the expression of cyclin E and CDK6 in p53+/+ or p53−/− HCT116 cells (Figure 7C,D).

### 2.7. Fucoidan Increases the Expression of CDK Inhibitors (CDKI) in HCT116 Cells

**Figure 8:**
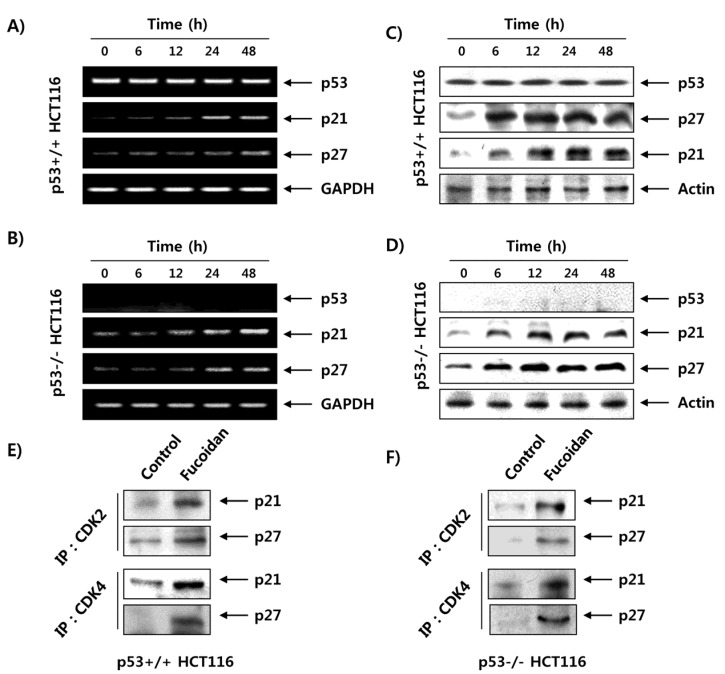
Induction of CDKIs and enhanced association of CDKIs with CDKs by fucoidan in HCT116 cells. (**A**,**B**) After treatment with 150 μg/mL of fucoidan for the indicated times, total RNA was isolated and reverse transcribed. The resulting cDNAs were then subjected to PCR with the indicated primers, and the reaction products were separated on 1.5% agarose gel and visualized by EtBr staining. (**C**,**D**) The cell lysates were prepared, and equal amounts of total cell lysates were subjected to SDS–PAGE, transferred, and probed with the indicated antibodies. GAPDH and actin were used as internal controls for the reverse transcription (RT)–PCR and Western blot assays, respectively. (**E**,**F**) The cells were treated with 150 μg/mL of fucoidan for 24 h, and then total cell lysates (500 μg) were prepared and immunoprecipitated with the anti-CDK2 or anti-CDK4 antibodies. The immunocomplexes were separated on 12% SDS–polyacrylamide gels, transferred onto membranes, probed with anti-p21 or anti-p27 antibodies, and visualized using an ECL detection system.

Contrary to the role of CDKs and cyclins, the CDKIs, including p21WAF1/CIP1 and p27KIP1, trigger cell cycle arrest by binding to cyclin/CDK complexes to inhibit their catalytic activity [29,30]. Therefore, we further investigated the effects of fucoidan on CDKI expression. As indicated in Figure 8A,B, p21 and p27 were commonly upregulated in mRNA following fucoidan treatment in p53+/+ and p53−/− HCT116 cells. On the other hand, the level of p53 tumor suppressor in p53 cells was not changed after the same treatment, and p53 was not detected in p53−/− HCT116 cells. Although p21 is a critical transcriptional target of p53 to mediate DNA damage-induced cell cycle arrest, our data showed that p21 expression was increased by fucoidan treatment, regardless of the p53 status in HCT116 cells, indicating that fucoidan regulates p21 expression in a p53-independent manner. The protein levels of p21 and p27, like their mRNA expression, were also gradually increased following fucoidan treatment in both cell lines (Figure 8C,D). In addition, the coimmunoprecipitation results showed that the bindings of CDK2 and CDK4 to p21 and p27 were markedly increased by treatment with fucoidan compared with those of control cells (Figure 8E,F); this was observed in both p53+/+ and p53−/− HCT116 cells.

## 3. Discussion

The tumor suppressor p53 is often critical for tumor suppression through the induction of apoptosis, cellular senescence, and cell cycle arrest [7,8]. Therefore, loss of p53 function is a major cause of tumor resistance to chemotherapeutic agents with a higher rate of proliferation and a more aggressive phenotype than similar tumors with wild-type p53 [12,31]. Thus, extensive efforts have been directed to the discovery of drugs that exhibit p53-independent action. In the current study, we investigated the role of p53 in the antiproliferative activity induced by fucoidan on two p53 isogenic HCT116 cell lines. The results obtained in the present study showed that treatment with fucoidan similarly reduced the cell viability of p53+/+ and p53−/− HCT116 cell lines (Figure 1), which was related with apoptosis induction that was characterized by chromatin condensation, DNA fragmentation, sub-G1 phase cell accumulation, and PARP cleavage (Figure 2, Figure 3 and Figure 4). The observed apoptotic induction in both HCT116 cell lines obviously resulted from DNA damage, as reflected by the increased phosphorylation of γH2AX, a double-stranded DNA breakage marker (Figure 4). These data collectively suggest that the proliferation-inhibitory effect of fucoidan is related with apoptosis induction and DNA damage, and this event was independent of p53 expression in HCT116 cells.

In addition to apoptosis, inhibition of cell cycle progression is an important strategy to control cancer cell growth, and the induction of cell cycle arrest by chemopreventive agents could be an effective approach in treating uncontrolled cell proliferation and survival in tumor cells. Therefore, a considerable number of natural compounds targeting cell cycle regulatory factors have been investigated as candidate treatments for attenuating the proliferation of cancer cells [32]. In eukaryotic cells, the cell cycle is strictly regulated by positive and negative growth-regulatory signals mediated by a series of cell cycle regulators, such as cyclins, CDKs, and CDKIs. Cyclin molecules, which act at different check points of the cell cycle, regulate the progression of each phase of the cell cycle by associating with corresponding phase-specific CDKs [14,33]. According to the results of the cell cycle analysis in this study, the inhibition of proliferation of HCT116 cells by fucoidan treatment was associated with G1 phase arrest in the cell cycle, regardless of the presence of wild-type p53 (Figure 5), indicating that G1 cell cycle arrest induced by fucoidan occurred through a p53-independent mechanism. These results are consistent with previous reports demonstrating that fucoidan triggers apoptosis and G1 cell cycle arrest in leukemia cells and bladder cancer cells, respectively, without any change in p53 expression [34,35].

During cell cycle progression from the G1 to the S phase, D-type cyclins activate CDK4 and CDK6, which assist in the maintenance and progression of the early G1 phase of the cell cycle. Moreover, cyclin E associates with CDK2 and plays a critical role in the transition from the G1 to S phase of the cell cycle [33,36]. During the G1/S transition, the activated CDKs phosphorylate and dissociate pRB, a tumor suppressor protein, from transcription factor E2Fs. This leads to the liberation of the E2Fs, which then activate a transcription program required for DNA synthesis, S-phase progression, and cell division [27,28]. Contrarily, CDKIs bind to CDK/cyclin complexes and suppress their kinase activity, thereby inducing hypophosphorylated pRB to sequestrate E2Fs. This inhibits E2Fs’ ability to carry out the cell cycle [29,37]. In this context, increasing the expression of CDKIs has been attempted for induction of growth arrest in most cancer cells. In this study, fucoidan induced consequent dephosphorylation of pRB, resulting in enhanced interaction between pRB and E2Fs, such as E2F-1 and E2F-4, in p53+/+ and p53−/− HCT116 cells (Figure 6). The data suggest that fucoidan represses the transcriptional activity of E2Fs for S-phase entry by promoting their binding with pRB in HCT116 cells. In addition, although expressions of cyclin D1, CDK2 and CDK4 were partially inhibited, they were still expressed to some extent until 48 h after treatment with fucoidan in the two HCT116 cell lines, and there was no significant change in expression of cyclin E or CDK6 in terms of either mRNA or protein levels (Figure 7). However, regardless of p53 status, expressions of p21 and p27—members of the Cip/Kip family—at both the transcriptional and translational levels were time-dependently increased in the two HCT116 cell lines in response to fucoidan (Figure 8).

Although p21 was first reported to induce cell cycle arrest due to an increase in p53-dependent transcriptional activity, it has been reported that many anticancer agents may increase the transcriptional activity of p21 in a p53-dependent manner [8,30]. Thus, no change in p53 expression was observed in wild-type p53 HCT116 cells treated with fucoidan, suggesting that fucoidan enhanced the transcriptional activity of p21 in a p53-independent manner. Our coimmunoprecipitation results also demonstrated that there were no detectable complexes between p21 with CDK2 and CDK4 or between p27 with CDK2 and CDK4 in untreated control cells; however, fucoidan treatment resulted in a marked increase in the binding of p21 and p27 with CDK2 and CDK4 in p53+/+ and p53−/− HCT116 cells (Figure 8). These data suggest that fucoidan’s capacity to arrest HCT116 cells in the G1 phase may be linked to the inhibition of CDK activity through binding of the CDK inhibitor proteins p21 and p27 to the cyclin/CDK complexes rather than by altering the levels of G1 phase–associated cyclins and CDKs proteins, and that the effects are independent of p53 status.

## 4. Materials and Methods

### 4.1. Cell Culture and Fucoidan Treatment

p53+/+ and p53−/− HCT116 cells were kindly provided by Dr. Bert Vogelstein (Johns Hopkins University, Baltimore, MD, USA) and grown at 37 °C in a humidified incubator under 5% CO_2_ in Roswell Park Memorial Institute 1640 medium (WelGENE, Daegu, Korea) supplemented with 10% fetal bovine serum and 1% penicillin-streptomycin (WelGENE). Fucoidan was purchased from Sigma-Aldrich Chemical Co. (Product F5631, isolated from *Fucus vesiculosus*, St. Louis, MO, USA) and dissolved in phosphate-buffered saline (PBS) as a stock solution at a concentration of 200 mg/mL; the stock solution was then diluted with the medium to the desired concentrations prior to use.

### 4.2. Cell Viability Assay

For the cell viability assay, the MTT reduction assay was used as described previously [38]. In brief, the cells were treated with various concentrations of fucoidan for 24 h or 48 h, and MTT working solution (0.5 mg/mL, Sigma-Aldrich Chemical Co.) was added to the culture media. After further incubation at 37 °C for 2 h, the supernatant was removed from the wells, and dimethyl sulfoxide (DMSO, Sigma-Aldrich Chemical Co.) was added to completely dissolve the formazan crystals. The absorbance of each well was measured at a wavelength of 590 nm using an enzyme-linked immunosorbent assay reader (Molecular Devices, Sunnyvale, CA, USA). At the end of incubation, cells from each well were also photographed under a phase-contrast microscope (Carl Zeiss, Oberkochen, Germany).

### 4.3. Cell Cycle Analysis

After treatment with fucoidan, the cells were harvested with trypsin and washed twice with cold PBS; following this, a DNA reagent kit (Cycle TEST™ PLUS kit, Becton-Dickinson, San Jose, CA, USA) was used according to the manufacturer’s instructions to stain the nucleus. Thereafter, flow cytometry analysis was performed using a flow cytometer (Becton-Dickinson), and the relative DNA content was determined using Cell Quest software (Becton-Dickinson) based on the amount of red fluorescence.

### 4.4. Nuclear Staining with DAPI

After treating the cells with fucoidan, the cells were fixed with 3.7% paraformaldehyde (Sigma-Aldrich Chemical Co.) in PBS for 15 min at room temperature. The fixed cells were washed once with PBS and attached on a slide glass using cytospin (Shandon, Pittsburgh, PA, USA). After staining with DAPI (Sigma-Aldrich Chemical Co.) solution (2.5 µg/mL) for 20 min in the dark at room temperature, the cells were washed twice with PBS and observed under a fluorescence microscope for analysis (Carl Zeiss).

### 4.5. DNA Fragmentation Assay

The fragmented DNA was selectively extracted using a Suicide-Track™ DNA Ladder Isolation kit (Calbiochem, San Diego, CA, USA) according to the manufacturer’s instructions. For detecting the DNA ladder, the extracted DNA samples were separated on a 1.5% agarose gel in Tris-acetic acid-ethylenediaminetetraacetic acid (EDTA) buffer at 100 V. After electrophoresis, the gel was stained with 0.1 μg/mL EtBr (Sigma-Aldrich Chemical Co.), visualized under ultraviolet light, and photographed.

### 4.6. Protein Isolation, Immunoprecipitation, and Western Blot Analysis

Cellular proteins were extracted using a lysis buffer (50 mM Tris-HCl [pH 7.4], 150 mM NaCl, 1% NP-40, 1% Triton X-100, 0.25% sodium deoxycholate, 1 mM EDTA, 10% glycerol) containing a complete protease inhibitor cocktail tablet (Roche Diagnostics, Mannheim, Germany). Protein concentrations were quantified using a Bio-Rad protein assay (Bio-Rad Laboratories, Hercules, CA, USA), according to the manufacturer’s instructions. For Western blot analysis, equal amounts of protein (30–50 μg) were separated by SDS–PAGE and transferred to polyvinylidene difluoride membranes (Schleicher & Schuell, Keene, NH, USA) by electroblotting. The blots were blocked with Tris-buffered saline (10 mM Tris-Cl, pH 7.4) containing 0.5% Tween-20 and 5% nonfat dry milk for 1 h at room temperature and then probed with the indicated primary antibodies. After overnight incubation, PBS containing 0.1% Tween 20 was used to wash the membranes twice, before and after incubation, with the secondary antibodies conjugated to horseradish peroxidase (Amersham Co., Arlington Heights, IL, USA) for 1 h at room temperature. Immunoreactivity was detected using the ECL solution kit (Amersham Co.) according to the manufacturer’s recommended protocol [39].

### 4.7. RNA Isolation and RT–PCR Analysis

Cells were harvested, and total RNA was extracted using TRIzol reagent (Invitrogen Life Technologies, Carlsbad, CA, USA) according to the manufacturer’s instructions. cDNA was synthesized from RNA and reverse transcribed using AccuPower® RT PreMix (Bioneer, Daejeon, Korea) containing Moloney murine leukemia virus reverse transcriptase. PCR was then carried out in a Mastercycler (Eppendorf, Hamburg, Germany) using gene-specific primers (Bioneer). The sequences of the primers were as follows: for cyclin D1, 5′-TGG ATG CTG GAG GTC TGC GAG GAA-3′ (sense) and 5′-GGC TTC GAT CTG CTC CTG GCA GGC-3′ (antisense); for cyclin E, 5′-AGT TCT CGG CTC GCT CCA GGA AGA-3′ (sense) and 5′-TCT TGT GTC GCC ATA TAC CGG TCA-3′ (antisense); for CDK2, 5′-GCT TTC TGC CAT TCT CAT CG-3′ (sense) and 5′-GTC CCC AGA GTC CGA AAG AT-3′ (antisense); for CDK4, 5′-ACG GGT GTA AGT GCC ATC TG-3′ (sense) and 5′-TGG TGT CGG TGC CTA TGG GA-3′ (antisense); CDK6, 5′-CGA ATG CGT GGC GGA GAT C-3′ (sense) and 5′-CCA CTG AGG TTA GAG CCA TC-3′ (antisense); for p53, 5′-GCT CTG ACT GTA CCA CCA TCC-3′ (sense) and 5′-CTC TCG GAA CAT CTC GAA GCG-3′ (antisense); for p21, 5′-CTC AGA GGA GGC GCC ATG-3′ (sense) and GGG CGG ATT AGG GCT TCC-3′ (antisense); for p27, 5′-AAG CAC TGC CGG GAT ATG GA-3′ (sense) and 5′-AAC CCA GCC TGA TTG TCT GAC-3′ (antisense); for glyceraldehyde 3-phosphate dehydrogenase (GAPDH), 5′-CGG AGT CAA CGG ATT TGG TCG TAT-3′ (sense) and 5′-AGC CTT CTC CAT GGT GGT GAA GAC-3′ (antisense). The PCR reaction was initiated at 94 °C for 2 min, followed by 31 cycles of 94 °C for 30 s, annealing temperature, 72 °C for 30 s, and a final extension step at 72 °C for 5 min. The amplified products were electrophoretically separated on a 1.5% agarose gel containing EtBr and visualized [40]. In a parallel experiment, GAPDH was used as an internal control.

### 4.8. Coimmunoprecipitation Analysis

Coimmunoprecipitation analysis was performed as described previously [41]. In brief, 500 μg of cellular proteins were incubated with immunoprecipitating antibodies in extraction buffer for 6 h at 4 °C. The immunocomplexes were then precipitated with protein A-Sepharose beads (Sigma-Aldrich Chemical Co.) for a further 2 h for precipitation. Beads were then washed twice with buffer and three times with cold PBS; following this, they were resuspended in 5X sample buffer. The eluted proteins were subsequently analyzed by SDS–PAGE.

### 4.9. Statistical Analysis

Each result is expressed as the mean ± SD of data obtained from triplicate experiments. A paired Student *t*-test was used for statistical analysis. Differences less than *p* < 0.05 were considered statistically significant.

## 5. Conclusions

Overall, the present data confirmed the anticancer activity of fucoidan in HCT116 CRC cells. We studied this process in HCT116 cell lines differing only in their p53 status, and we did not observe any difference in fucoidan-induced apoptosis and G1 cell cycle arrest based on the p53 status of the cells, suggesting that these events are independent of p53 status. As p53 mutation is frequently found in CRC, the development of chemotherapeutics that are still effective in p53 mutant tumors is a critical issue for CRC therapy. If further evidence proves the anticancer properties of fucoidan in CRCs with dysregulation of Wnt/β-catenin signaling and Ras signaling, the general signaling pathways frequently mutated in CRC, fucoidan could be suggested as a prospective novel therapeutic for CRC.
